# MicroRNA-mediated metabolic regulation of immune cells in cancer: an updated review

**DOI:** 10.3389/fimmu.2024.1424909

**Published:** 2024-06-28

**Authors:** Sepideh Chowdary Khameneh, Sara Razi, Ramin Lashanizadegan, Sanaz Akbari, Masoud Sayaf, Karimeh Haghani, Salar Bakhtiyari

**Affiliations:** ^1^ Vira Ideators of Modern Science, Tehran, Iran; ^2^ Vira Pioneers of Modern Science (VIPOMS), Tehran, Iran; ^3^ Nourdanesh Institute, Isfahan, Iran; ^4^ Department of Cellular and Molecular Biology, Faculty of Basic Sciences, Azad University Central Tehran Branch, Tehran, Iran; ^5^ Department of Clinical Biochemistry, School of Medicine, Ilam University of Medical Sciences, Ilam, Iran; ^6^ Feinberg Cardiovascular and Renal Research Institute, Northwestern University School of Medicine, Chicago, IL, United States

**Keywords:** microRNAs (miRNAs), metabolic regulation, immune cells, cancer, immunotherapy

## Abstract

The study of immunometabolism, which examines how immune cells regulate their metabolism to maintain optimal performance, has become an important area of focus in cancer immunology. Recent advancements in this field have highlighted the intricate connection between metabolism and immune cell function, emphasizing the need for further research. MicroRNAs (miRNAs) have gained attention for their ability to post-transcriptionally regulate gene expression and impact various biological processes, including immune function and cancer progression. While the role of miRNAs in immunometabolism is still being explored, recent studies have demonstrated their significant influence on the metabolic activity of immune cells, such as macrophages, T cells, B cells, and dendritic cells, particularly in cancer contexts. Disrupted immune cell metabolism is a hallmark of cancer progression, and miRNAs have been linked to this process. Understanding the precise impact of miRNAs on immune cell metabolism in cancer is essential for the development of immunotherapeutic approaches. Targeting miRNAs may hold potential for creating groundbreaking cancer immunotherapies to reshape the tumor environment and improve treatment outcomes. In summary, the recognition of miRNAs as key regulators of immune cell metabolism across various cancers offers promising potential for refining cancer immunotherapies. Further investigation into how miRNAs affect immune cell metabolism could identify novel therapeutic targets and lead to the development of innovative cancer immunotherapies.

## 1 Introduction

Immunometabolism is a field of study that focuses on how immune cells manage their metabolism to sustain optimal function. This involves understanding the intricate connection between cellular metabolism and immune cell performance, particularly in the context of diseases such as cancer. In essence, immunometabolism explores how the metabolic activity of immune cells influences their activation and functionality, and how this, in turn, impacts disease progression and treatment outcomes (1, 2). It is now understood that immunological signals can also trigger changes in the core metabolic pathways inside immune cells, in addition to dietary factors and oxygen levels. Specific metabolic pathways may affect immune cell phenotype and function and their roles in energy production and biosynthesis (1).

Cancer contributes significantly to mortality rates and presents a formidable challenge to global initiatives aimed at extending human lifespan (3). Various therapeutic approaches have been developed to tackle cancer or circumvent drug resistance. Cancer cells frequently undergo extensive and intricate metabolic reprogramming to fulfill the biosynthetic and bioenergetic demands of growth and adaptability to the “stressful” tumor microenvironment (TME). The TME is influenced by several factors, including the Warburg effect, in which cancer cells preferentially rely on glycolysis for ATP production; hypoxia; and changes in pH. The metabolic activity of tumor cells may impact the immune system by producing metabolic by-products that significantly affect immune cell activation, fitness, and effector function, or by competing fiercely for vital resources (such as glucose, glutamine, lipids, and amino acids) (4, 5). Consequently, these dysfunctional immune cells are not only unable to eliminate cancer cells, but also have the potential to transform into cells that promote uncontrolled cell division, facilitating the spread of the tumor (6, 7).

The percentage of the mammalian genome that is transcribed is substantial, with over 80% of the genome being expressed. Furthermore, it is worth noting that only a small fraction, less than 3%, of these sequences encode protein-coding genes. These findings suggest that the non-coding component of the genome may play a role in physiological and pathological processes (8). MicroRNAs (miRNAs) have been the subject of extensive research as a class of non-coding RNAs in cancer (9, 10). MiRNAs are short RNA molecules (20 to 22 nucleotides in length) and act as negative regulators of the transcription process by binding to the 3′-UTRs of their target mRNAs and facilitating their degradation. Over 2000 miRNAs have been identified in the human genome, with the potential to influence more than 60% of the protein-coding genes (11). MiRNAs also play a role in regulating various dysregulated biological processes in cancer, such as differentiation, proliferation, and apoptosis, as well as interactions between malignant cells and the tumor microenvironment (TME) (12, 13). The potential use of miRNAs as therapeutic tools and targets in the biology of primary childhood tumors has received increasing attention (14). Depending on the function of their targets, miRNAs can act as either oncogenes or tumor, suppressors. Onco-miRNAs are often upregulated in tumors and target tumor-suppressor genes, whereas in malignancies, tumor-suppressor miRNAs are downregulated, leading to upregulation of their respective oncogenic targets (15). Katoh et al. reviewed several miRNAs that can act on both sides as onco-miRNAs and tumor suppressor miRNAs, including miR-24, miR-125b, miR-195, and miR-214 respectively in acute myocardial infarction, ischemia/reperfusion injury, cardiomyopathy, cardiac ischemia and heart failure (16). One mechanism by which miRNAs are involved in the immune system in cancer is through metabolic pathways in the immune cells. However, recent studies have shown that miRNAs also play crucial roles in the metabolic regulation of immune cells in malignancies (17). In this review, we highlight new perspectives on the significant role of miRNAs in the metabolic regulation of immune cells in different malignancies.

## 2 Role of miRNAs in regulating immune cell subsets and functions

MicroRNAs play a crucial role in regulating immune functions by directing the development of specific immune cell subsets, such as B cells and T cells. It has been shown that miRNAs regulate the development and function of various immune cell subsets, including T cells, B cells, and natural killer (NK) cells (18–20). For instance, miRNAs modulate T cell differentiation and functionality by regulating genes critical for T-cell receptor (TCR) and cytokine receptor signaling (21). Similarly, miRNAs can regulate the differentiation and function of B cells by controlling the expression of genes involved in B cell receptor signaling and antibody production. The complexity of the role of miRNAs in immune regulation involves multiple genes and signaling pathways. The miR-17–92 cluster is particularly crucial for B cell development; mice deficient in miR-17–92 do not survive post-birth due to lung hypoplasia and ventricular septal defects, linked to elevated levels of the pro-apoptotic protein Bim that hinder B-cell growth (22). Additionally, increased miR-17–92 expression in lymphocytes predisposes mice to lymphoproliferative diseases and autoimmunity, characterized by enhanced lymphocyte proliferation and reduced activation-induced cell death, primarily through the downregulation of PTEN and BIM, contributing to lymphoma formation (23). MiR-124 plays a significant role in both adaptive and innate immune responses and is overexpressed in immune-centric tissues such as bone marrow, lymph nodes, and thymus. Studies indicate that miR-124 expression is low in human cord blood CD34(+) cells but increases during cell differentiation (22).

In the context of multiple sclerosis, Guerau-de-Arellano and colleagues demonstrated that overexpression of miR-128 and miR-27b in naive CD4(+) T cells, and miR-340 in memory CD4(+) T cells, leads to the suppression of BMI1 and IL4, reduction in GATA3 levels, and a cytokine shift from Th2 to Th1 phenotype. Pharmacological inhibition of these miRNAs was shown to restore the Th2 response, highlighting the critical role of miRNAs in modulating T-cell phenotypes in autoimmune diseases (24).

T regulatory cells (Tregs) are essential for maintaining immune homeostasis, peripheral tolerance, and suppressing excessive immune responses. Dysregulation or overactivity of Tregs can trigger various immune-related disorders and cancers (25). Microarray studies have identified specific miRNA profiles in Tregs in both mice and humans, with the initial discovery of the human nTreg miRNA signature, comprising differentially expressed miRNAs such as miR-21, 31, 125a, 181c, and 374, by Rouas et al. (26). Sadlon et al. highlighted miRNAs regulated by Foxp3, identifying distinct expressions of miR-146a, miR-155, hsa-let7, miR-101, miR-7, and miR-142–5p in nTregs compared to Th cells, with a noted downregulation of miR-19b and miR-20b (27).

## 3 Role of miRNAs in tumor cell responses

Cancer is widely regarded as one of the most intricate diseases. In a comprehensive review, Hanahan et al. outlined several prominent characteristics of cancer, including the preservation of proliferative signals, invasion and metastasis, induction of angiogenesis, resistance to cell death, and evasion of immune system damage (22, 24). Additionally, in cancer biology, the relationship between genetic and epigenetic changes is important (24, 25). Glioblastoma (GBM), the most common form of primary high-grade brain tumor, exhibits an extremely poor survival rate (26). GBM serves as an example of a complex tumor environment (TME), in which interactions between cancer and stromal cells alter the immunometabolism of the tumor. Metabolic changes associated with GBM, including the mutation status of isocitrate dehydrogenases that involve enzymatic modifications, are crucial for initiating Glioma CpG Island Methylation phenotypes, ultimately resulting in epigenetic alterations (27). In a well-known article, they investigated the potential consequences of genomic instability, including epigenetic modifications, on metabolic reprogramming and immunosuppression in glioblastoma multiforme (GBM) (28).

Notably, over half of all miRNA-encoding genes are located in cancer-related genomic regions or fragile sites, implying a role for miRNAs in both these processes (29). Abnormal miRNA expression in cancerous tissues and cells has been linked to its involvement in the pathological mechanisms of cancer (30). In recent years, the scientific community has recognized the significance of miRNAs as essential players in various malignancies (31, 32). Yi et al. reported that miR-3662 has oncogenic properties in triple-negative breast cancers (TNBCs), promoting tumor progression and metastasis (33). Moreover, data from The Cancer Genome Atlas (TCGA) show that miR-3662 expression is higher in triple-negative breast tumors (TNBCs) compared to other types of breast (34). The deletion of miR-3662 in TNBC cells has been found to inhibit their migration, tumor invasion, and growth both *in vivo* and *in vitro*, according to additional research. CRISPR and dual-luciferase experiments have revealed that the transcription factor HBP-1 binds to miR-3662 and promotes the growth of TNBC cells. This suggests that dysregulation of miR-3662 may have significant therapeutic potential in TNBC patients (33). Another extensive study by Pan et al. revealed that miR-33a-5p and miR-128–3p play tumor-suppressive roles in lung cancer. The researchers utilized qRT-PCR analysis that revealed that these two miRNAs were significantly downregulated in the lung cancer tissues, cell lines, and whole blood samples. These findings suggest that these two miRNAs may be valuable biomarkers for lung cancer detection. Furthermore, the results from the blood samples indicated that miR-33a-5p and miR-128–3p could serve as minimally invasive markers for the early detection of lung cancer (35). Additionally, chemotherapy and radiation therapy employed in ovarian cancer treatment may influence the expression of miR-200c-3p and its target genes, such as PD-L1. miR-200c-3p concurrently downregulates the expression of PD-L1, c-Myc, and β-catenin, consequently heightening the susceptibility of ovarian cancer cells to olaparib and irradiation (36). Yuan et al. explored the role of miR-362–3p in the development of epithelial ovarian cancer. The levels of miR-362–3p were found to be significantly lower in tumor tissues and cell lines derived from ovarian cancer. Additionally, this study demonstrated that miR-362–3p impedes cell proliferation and migration by binding to the 3’-UTR of MyD88. These findings suggest that miR-362–3p has a tumor-suppressive effect in epithelial ovarian cancer (37).

According to Vychytilova-Faltejskova et al., the expression of miR-215–5p is lower in the tumor tissues of colorectal cancer patients than in the adjacent tissues. Low levels of this miRNA are correlated with metastasis, tumor stage, and shorter overall survival. This study revealed that miR-215–5p induces apoptosis in colorectal cancer cells and suppresses cell proliferation and migration. Furthermore, epiregulin (EREG) and homeobox B9 (HOXB9) were identified as target genes regulated by miR-215–5p. Overall, this study suggests that miR-215–5p, as a tumor suppressor, could be a valuable tool in the diagnosis and prognosis of colorectal cancer (38).

The expression levels of miR-671–5p were found to be significantly high in prostate cancer, as revealed by Zhu et al., and were associated with poor prognosis. Additionally, both *in vitro* and *in vivo* experiments have demonstrated that miR-671–5p plays a role in promoting prostate tumorigenesis and migration. Also they showed that miR-671–5p modulates nuclear factor I A (NFIA), thereby contributing to prostate tumorigenesis (39). In another study, Yang et al. discovered that miR-200a is significantly overexpressed in human bladder cancer tissues. They further found that miR-200a overexpression led to human bladder cancer invasion by upregulating matrix metalloproteinase (MMP)-2, inhibiting Dicer expression, and miR-16 maturation. These results indicate that miR-200a is an onco-miRNA that can be used to identify new treatment strategies for patients with invasive bladder cancer (40). Qian et al. (2017) also found that miR-26a and let-7a suppressed the proliferation and invasiveness of malignant melanoma cell lines. Moreover, miR-26a regulates microphthalmia-associated transcription factor (MITF) expression and potently promotes apoptosis. These findings suggest that miR-26a and let-7a may be potential therapeutic agents for malignant melanoma (41). Wan et al. (2020) assessed the impact of miR-324–5p upregulation on the growth of pancreatic cancer cells by targeting Krüppel-like factor 3 (KLF3), a transcriptional repressor. These results demonstrated that blocking miR-324–5p inhibited cell proliferation and induced cell death (42). Wang et al. (2018) investigated the role of miRNAs in cancer and found that miR-125b was downregulated in thyroid cancer tissue samples and cell lines. They showed that miR-125b regulates and inhibits the expression of Foxp3, which subsequently promotes autophagy and improves the effectiveness of cisplatin in thyroid cancer through the Atg7 pathway both *in vitro* and *in vivo* (43). Additionally, Takakura et al. (2008) reported that miR-19a, a member of the oncogenic miR-17–92 cluster, is overexpressed in anaplastic thyroid cancer, which is one of the most undifferentiated, invasive, and fatal types of thyroid cancer (44). Calabrese et al. (2018) found that upregulation of miR-19a promotes cell growth and affects the expression of genes involved in thyroid cell differentiation and invasion (45). Li et al. (2017) identified miR-3174 as the most significantly differentially expressed miRNA in gastric cancer by screening the TCGA dataset. This study demonstrated the aberrant expression of miR-3174 in gastric cancer tissues and cultured cell lines. Furthermore, in silico analyses and *in vitro* and *in vivo* experiments revealed that miR-3174 directly targeted Rho GTPase-activating protein 10 (ARHGAP10) and contributed to apoptosis and autophagic defects. Therefore, miR-3174 may be useful in the diagnosis and treatment of gastric cancer (46). Several studies have supported the hypothesis that miRNAs play a crucial role in carcinogenesis, and have emphasized the importance of these short ncRNAs in the detection, prognosis, and therapy of cancer.

## 4 The emerging role of microRNA in the control of immunometabolism

**Figure 1:**
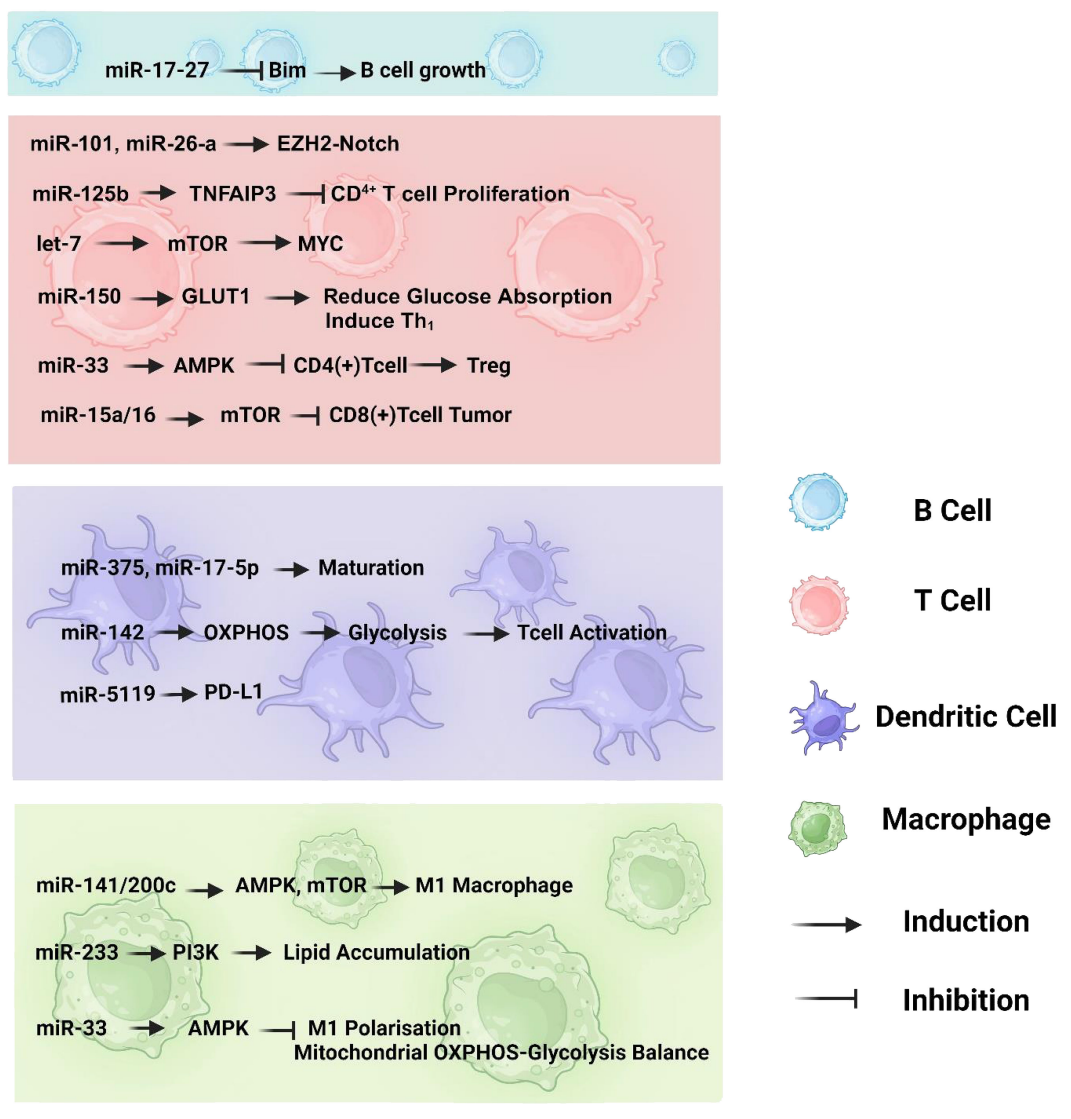
The involvement of miRNAs in the immunometabolism of various immune cell types, including B cells, T cells, dendritic cells, and macrophages. The pathways depicted highlight the crucial interplay between oxidative phosphorylation and glycolysis, as well as the impact of miRNAs on the maturation and differentiation processes of immune cells.

The involvement of miRNAs in immune cell regulation and metabolic reprogramming is well understood; miRNA-mediated metabolic control in immune cells remains primarily unexplored (47). Traces of miRNAs in the control of immunometabolism can be observed in T cells, B cells, macrophages and dendritic cells. In **Figure 1** we summarized microRNAs and their targeted pathways which regulate different immune cell metabolism in cancer development.

Naive, effector, and memory are the three main functional states of CD4+ and CD8+ T cells (48). Because of their unique bioenergetic requirements, it is impossible to prevent metabolic reprogramming during the transition between effector and memory T-cells. This requires energy, and the direction of metabolism towards anabolism is felt more in effector T cells due to the proliferation and secretion of cytokines than in memory T cells (49). Activated T cell effectors undergo metabolic reprogramming, shifting their cellular energy-production pathway from oxidative phosphorylation to aerobic glycolysis. In contrast, regulatory T cells (Tregs) and memory T cells undergo metabolic reprogramming that increases Fatty Acid Oxidation (FAO) while decreasing the need for glycolysis (50).

miRNAs can alter the immunometabolism of T cells by directly targeting the constituents of metabolic pathways, including metabolic enzymes and energy transporters, or by indirectly targeting some immune cell metabolic controllers, including adenosine monophosphate (AMP)-activated protein kinase-mammalian target of rapamycin (AMPK mTOR) and c-myc gene (Myc). One study found that miR-101 and miR-26a may target the enhancer of the zeste homolog 2 (EZH2)-Notch signaling pathway and decrease T cell aerobic glycolysis, thereby reducing tumorigenesis in ovarian cancer (51). In acute lymphoblastic leukemia, miR-125b specifically targets tumor necrosis factor-alpha-induced protein 3 (TNFAIP3), which inhibits CD4+ T cell proliferation, boosts glycolysis in T cells, and markedly increases oxygen consumption (52). These studies imply that miRNA-mediated metabolic reprogramming of T cells may improve cancer therapy. It has also been established that miR-150 directly targets GLUT1 in CD4+ T cells, thereby reducing glucose absorption and mediating Th1 activation. MiR-33 may also inhibit T-cell synthesis of Cpt1a, an enzyme that promotes mitochondrial FAO in the absence of CD28 (53). Thus, T cells undergo metabolic reprogramming to meet rising bioenergetic demands (54). It has been demonstrated that miR-33 can inhibit CD4+ T cell differentiation into Tregs by binding to AMPK, but knocking out miR-33 can boost Treg induction, demonstrating the substantial impact of miRNAs on the metabolic reprogramming of T cells by targeting essential metabolic moderators of immune cells (55). AMPK is responsible for T cell responses, which can be beneficial or detrimental. Although AMPK-dependent regulation of anabolic pathways, including the synthesis of proteins (mTORC1) and fatty acids (ACCa), inhibits effector T cell growth and function, AMPK is essential for the metabolic adaptability of such cells and is required to withstand the environmental challenges they experience throughout immune responses (56). Likewise, let-7 can potentially target mTOR and cause differentiation of T cells between stimulation and response failure. Furthermore, let-7 markedly affected the metabolic switch in stimulated CD8+ T cells by targeting Myc (57, 58). Myc may play a role in lymphocyte metabolic reprogramming, from oxidative phosphorylation to glycolysis (57). Another study on glioblastoma suggested that miR-15a/16 may inhibit the stimulation of tumor-infiltrating CD8+ T cells by targeting mTOR. As a result, the tumor-suppressive function is altered in the brain, leading researchers to hypothesize that miR-15a/16 may be a possible therapeutic target for brain cancer immunotherapy (40). It has been shown that miR-99a, in collaboration with miR-150, may suppress mTOR expression and enhance Treg development, providing a rationale for the involvement of these two miRNAs in T-cell differentiation (59).

Compared to T cells, the metabolism of B cells is less well documented; however, these lymphocytes have numerous similarities regarding metabolic reprogramming and/or available transition. Along the same lines as T cells, B cells can be divided into three functional states: naive, effector, and memory. Additionally, metabolic reprogramming is required for B cells during the transition between states (2). When B cells transition from a quiescent to a stimulated state, they significantly increase their need for energy, which is met by an increase in their capacity for glucose absorption and the rate at which they undergo aerobic glycolysis (60).

Classically activated macrophages (M1) and alternatively activated macrophages (M2) are two of the most common phenotypically unique subsets of macrophages. These subsets are characterized by substantial changes in gene expression and macrophage polarization (61). The progression of macrophage polarization is strongly influenced by metabolic reprogramming (62, 63). Metabolic reprogramming is necessary to supply the energy needed for macrophage activation, cell development, and function because of their high energy demands (64). Energy demands increase in M1 to preserve antigen-presenting capacity and rapid growth in response to pathogens or antigen stimuli; therefore, the cell undergoes metabolic remodeling to shift from oxidative phosphorylation to glycolysis. This allows for the rapid synthesis of substantially more energy (65). Unlike metabolically dormant M2, which generates most of its ATP through fatty acid oxidation (FAO), metabolically active M1 rapidly supplies the energy needed for the pro-inflammatory response by utilizing aerobic glycolysis (62, 64). miRNAs may also control macrophage metabolic reprogramming by explicitly targeting important immune cell metabolic regulators, such as AMPK, PI3K, and mTOR. Ouimet et al. discovered that miR-33 may control macrophage inflammatory polarization and disturb the balance between mitochondrial oxidative phosphorylation and aerobic glycolysis by targeting AMPK as an energy sensor (55). Subsequent research revealed that Mycobacterium tuberculosis-induced miR-33 expression might hinder mitochondrial FAO and cause the production and storage of higher cellular lipid accumulation in macrophages by targeting the AMPK pathway (66). Further research has revealed that miR-223 might prevent macrophage lipid accumulation, which in turn prevents the onset of atherosclerosis by activating the PI3K pathway (67). This is consistent with the notion of Tran et al., who reported that miR-141/200c regulates macrophage polarization by reprogramming various signal transduction pathways, particularly AMPK and mTOR. Accordingly, miR-141/200c depletion induced macrophage polarization to obtain the M2 phenotype. Previous studies have highlighted the significance of miRNA-associated regulation of metabolic pathways in macrophages via critical signaling pathways. Future studies should clarify how miRNAs in macrophages regulate metabolism, as these are still mostly unknown (68).

MiRNAs play a crucial role in regulating the metabolism of dendritic cells (DCs). miRNAs have been found to control the development, differentiation, and functions of DCs, making them important regulators of immune responses (69). Specifically, miR-142 is essential for the metabolic reprogramming of DCs from oxidative phosphorylation (OXPHOS) to glycolysis, which is necessary for an immunogenic response. In the absence of miR-142, DCs fail to switch to glycolysis and exhibit reduced production of proinflammatory cytokines and impaired T cell activation (70). Additionally, Furthermore, miRNA-5119 has been identified as a potential regulator of PD-L1 in DCs, and its mimic-engineered DC vaccines have shown promising results in enhancing anti-tumor immune responses in a mouse model of breast cancer (54, 71). Another study showed that Helicobacter pylori via down-regulating miR-375 to inhibit dendritic cell maturation resulting in gastric cancer. Also Cui Z et al. reported that miR-17–5p inhibits dendritic cell maturation in gastric cancer (72, 73).

## 5 Role of microRNA in cancer immunotherapy

MiRNAs are potent tumor suppressors and oncogenes with particular cancer-related activities that have the inherent capacity to influence the outcome of traditional therapy (74). Because they control how immune cells function and how the immune system responds, targeting miRNAs with miRNA-based therapies for cancer could improve immunotherapy together with other treatments. Using various techniques, miRNA as an immunotherapeutic agent is now being studied in preclinical trials. These immunotherapeutic targets are classified as either miRNA mimics or antagonists (71). MiRNA mimics are known to restore tumor suppressor miRNAs, primarily dedicated to immune checkpoint blockade, while miRNA antagonists work the same way as inhibitors. Direct improvements in tumor immunogenicity and increased sensitivity to standard treatments may result from miRNA targeting (74). **Table 1** provides brief and direct details of miRNAs involved in cancer immunotherapy.

**Table 1:** The target miRNAs involved in cancer immunotherapy.

Mechanism of action through cancer immunotherapy	Target miRNAs	Target genes	Type of cancer	References
**Mimic**	MiR-34a	PD-L1	Acute myelogenous leukemia	(75)
	MiR-34a	PD-L1	Non-small-cell lung carcinoma	(76)
	MiR-34a	PD-L1	Lymphoma	(77)
	Mir-424	PD-L1	Ovarian cancer	(78)
	MiR-138	CTLA-4 and PD-1	Glioma	(79)
	MiR-124	STAT3	Glioma	(80)
**Antagonist**	MiR-155	IL-6	Lung cancer	(81)
	MiR-23a	BLIMP-1	Melanoma	(82)
	MiR-17	EGFRvIII	Glioblastoma	(23)
	MiR-19b	Foxp3	Glioblastoma	(27)
	MiR-153	EGFR	Colon cancer	(83)
	MiR-143	HER2	Esophagus cancer	(76)
	MiR-200c	PD-L1	Ovarian cancer	(36)
	MiR-24	ICOSL	Lymphoma	(84)

### 5.1 MicroRNA mimics in cancer immunotherapy

Over the past decade, miRNA mimics, with miR-34a, miR-124, miR-424, and miR-138 as the primary miRNAs, has emerged as a pivotal target for immunotherapy (85). MiR-RX34 (MRX34), a miR-34a mimic, was the pioneering miRNA-based anticancer therapeutic tested in clinical trials (86). Another study highlighted the significance of PD-L1 inhibition and miR-34a dysregulation in immunotherapy, suggesting potential implications for the treatment of EBV-associated cancers (77). Also miR-34a was discovered to downregulate programmed death-ligand 1 (PD-L1) in acute myelogenous leukemia (AML) by targeting its mRNA (75). MiR-34a mimics may also have the potential to modulate immune cell subsets infiltrating tumor tissue. For instance, treatment of a mouse model of non-small cell lung cancer (NSCLC) with MRX34 increased tumor infiltration of cytotoxic (CD8+) T cells and decreased CD8+PD1+ T cells while downregulating PD-L1. Additionally, the number of radiation-induced macrophages and Treg cells is reduced, whereas CD8+ T cell infiltration is increased when MRX34 is administered alongside radiotherapy (XRT) (17). These findings highlight the enhanced effectiveness of an miR-34a mimic in modulating antitumor immune responses and controlling tumor development when combined with XRT (61). Mir-424 is also one of the miRNAs that targets PD-L1 and downregulates it *in vivo*. Disruption of PD-L1/PD-1 and CD80/CTLA-4 immune checkpoint signaling, as well as chemoresistance reversal mediated by miR-424 restoration, results in a synergistic effect that drives the escalating proliferation of specific CD8+T cells, causing an increase in the survival of a mouse model of ovarian cancer by decreasing myeloid-derived suppressive and regulatory T cells (78). In contrast, Foxp3 expression in T helper (CD4+ T) cells is regulated by miR-138, leading to downregulation of CTLA-4 and PD-1. Treg cell infiltration was found to be dramatically reduced after *in vivo* administration of a mouse glioma model with an miR-138 mimic, which suppressed the expression of CTLA-4, PD-1, and Foxp3 in tumor-infiltrating CD4+T cells (79). MiR-197, miR-513, and miR-570 are among the miRNAs that inhibit PD-L1 and may be appropriate targets for immunotherapy (87).

### 5.2 MicroRNA antagonists in cancer immunotherapy

Stronger and more efficient antitumor immune responses can be achieved by inhibiting miRNAs, which normally decrease immune cell activity. This idea merits additional investigation in light of the recent discovery of miRNA antagonists (88). *In vivo* tumor growth and metastasis are suppressed when miR-155 is inhibited by the systemic administration of corresponding anti-miRNA sequences (24). Since the lack of miR-155 in immune cells reduces their activity, systemic suppression of miR-155 could have varying effects on immune cell function. However, upregulation of miR-155 in tumor-specific CD8+ T cells may circumvent the possible adverse effects on immune responses driven by systemic injection of miR-155 inhibitors, leading to enhanced T-cell-based adaptive treatment (81). In addition, targeting the signal transducer and activator of transcription 3 (STAT3) by miR-124 is a crucial mechanism mediating immunosuppression in the TME. In this context, pro-immunogenic mediators interferon-gamma (IFN-γ), tumor necrosis factor-alpha (TNF-α), and interleukin-2 (IL-2) have been shown to be upregulated in glioma surroundings after injection of miR-124 mimics, leading to a significant anti-glioma therapeutic impact (80). The EBV may evade the host’s immune system by utilizing EBNA2. One mechanism it employs is the induction of miR-24 to diminish the expression of ICOSL. Leopizzi et al. showed that the use of inhibitors of miR-24 reconstituted the expression of ICOSL and enhanced the anti-cancer immune response against the EBNA2-transfected DLBCL line (84). By altering the expression of BLIMP-1, a transcription factor that regulates cell proliferation and cytotoxicity, miR-23a increases the toxicity of effector CD8+ CTLs. Tumor cells release TGF-β, which increases miR-23a levels. Tumor progression in a melanoma animal model was dramatically reduced by the adoptive transfer of CTL cells administered with miR-23a inhibitors (82). Additionally, miRNAs have been leveraged in adoptive cell therapy to enhance the efficiency of chimeric antigen receptor (CAR) T cells. Co-transduction of miR-17 and temozolomide (TMZ) enhances the lifespan and pharmacological efficacy of epidermal growth factor receptor variant III (EGFRvIII)-specific CAR T cells in GBM treatment. Similarly, miR-153 boosts the therapeutic efficacy of CAR T cells targeting EGFR in human colon cancer xenograft tumors (83). Furthermore, another study revealed that miR-143 enhances the development of central memory T cells and increases cytokine release. Further research revealed that the overexpression of miR-143 increased the specific killing activity of HER2-CAR T lymphocytes against TE-7 cells by reducing glucose absorption and glycolysis (76).

## 6 Conclusion

Further investigation is warranted to elucidate the role of miRNAs in regulating the metabolic processes of B cells, as changes in their metabolic state are intricately linked to alterations in functionality. It’s worth noting that miRNAs can directly and indirectly regulate immunometabolism in both B and T cells. Additionally, macrophages undergo significant metabolic changes due to modifications in primary signaling pathways involving miRNAs, although research in this area is still in its nascent stages. Therefore, more studies are needed to unravel the potential roles of miRNAs in regulating the metabolic processes of B cells, T cells, and macrophages, which will be pivotal in understanding the significance of miRNA-mediated regulation of immune cell metabolism in cancer development. For therapeutic applications, miRNA mimetics or inhibitors offer promising avenues for the treatment and prevention of cardiovascular diseases. The use of miRNAs in this field has great potential to revolutionize personalized medicine and enable treatments tailored to a person’s specific genetic makeup. Furthermore, due to their regulatory role in genes related to metabolic function, the prominence of miRNAs in metabolic disorder research is steadily increasing.

In addition to their crucial role in regulating immune cell metabolism, clinical trials focusing on microRNAs (miRNAs) hold significant importance due to their potential implications in the diagnosis, prognosis, and treatment of various diseases. For instance, clinical trials investigating cobomarsen in Diffuse Large B Cell Lymphoma (DLBCL) shed light on the therapeutic potential of miRNA-based interventions in cancer treatment. Similarly, studies such as NCT06325111, exploring the role of miRNAs in autoimmune diseases and metabolic disorders, underscore the broad spectrum of applications for miRNA-based therapies. By elucidating the mechanisms underlying miRNA dysregulation in different pathologies, these trials contribute invaluable insights into the development of targeted therapeutic strategies. Therefore, the exploration of miRNAs through clinical trials is pivotal not only for advancing our understanding of disease mechanisms but also for paving the way towards innovative therapeutic interventions tailored to specific patient populations (89, 90).

## Author contributions

SK: Investigation, Writing – original draft. SR: Writing – original draft, Software. RL: Software, Writing – original draft. SA: Data curation, Writing – original draft. MS: Formal analysis, Writing – original draft. KH: Conceptualization, Data curation, Formal analysis, Funding acquisition, Investigation, Methodology, Project administration, Resources, Software, Supervision, Validation, Visualization, Writing – original draft, Writing – review & editing. SB: Conceptualization, Data curation, Formal analysis, Funding acquisition, Investigation, Methodology, Project administration, Resources, Software, Supervision, Validation, Visualization, Writing – original draft, Writing – review & editing.

## Glossary

3′-UTRs: 3′-untranslated regions
5′-UTR: 5′-untranslated regions
Ago2: Argonaute 2
AML: Acute myelogenous leukemia
AMP: Adenosine monophosphate
AMPK mTOR: AMP-activated protein kinase-mammalian target of rapamycin
ARHGAP10: Rho GTPase Activating Protein 10
ATP: Adenosine triphosphate
BLIMP-1: B lymphocyte-induced maturation protein-1
BMI: B lymphoma Mo-MLV insertion region 1 homolog
Bregs: Regulatory B cells
CAR: Chimeric antigen receptor
CD80/CTLA-4: cluster of differentiation 80/cytotoxic T-lymphocyte-associated protein 4
CTLs: Cytotoxic T lymphocytes
EGFRvIII: epidermal growth factor receptor variant III
EREG: Epiregulin
EZH2: Enhancer of zeste homolog 2
FAO: Fatty Acid Oxidation
GLUT1: Glucose transporter 1
GBM: Glioblastoma
HBP-1: HMG-box transcription factor 1
HER2: Human epidermal growth factor receptor 2
HK2: Hexokinase 2
HOXB9: Homeobox B9
IDO: Indoleamine
IFN-γ: Interferon gamma
IL4: Interleukin-4
IL2: Interleukin-2
KLF3: Krüppel-like factor 3
miRNAs: MicroRNAs
MITF: Microphthalmia-associated transcription factor
MMP: Matrix metalloproteinase
MRX34: MiR-RX34
Myc: c-myc gene
MyD88: Myeloid differentiation primary response 88
NFIA: Nuclear factor I A
NSCLC: Non-small-cell lung carcinoma
PD-1: Programmed cell death protein 1
PD-L1: Programmed death-ligand 1
pre-miRNAs: Precursor miRNAs
pri-miRNAs: Primary miRNAs
PTEN: Phosphatase and tensin homolog
qRT-PCR: Quantitative real-time Reverse Transcription PCR
RNA Pol II: RNA polymerase II
STAT3: Signal transducer and activator of transcription 3
TCGA: The Cancer Genome Atlas
TCR: T-cell receptor
TGF-B: Transforming growth factor beta
TGFRII: Targeting transforming growth factor beta receptor 2
Th1: T-helper 1
Th2: T-helper 2
TME: Tumor microenvironment
TNBCs: Triple-negative breast cancers
TNF-α: Tumor Necrosis Factor-alpha
TNFAIP3: Tumor necrosis factor, alpha-induced protein 3
Tregs: Regulatory T cells
XRT: MRX34 with radiotherapy
